# Beyond algorithms: The human touch machine-generated titles for enhancing click-through rates on social media

**DOI:** 10.1371/journal.pone.0306639

**Published:** 2024-07-12

**Authors:** Wenyu Yang

**Affiliations:** Foki Media Co., Ltd. Hangzhou, Hangzhou, Zhejiang Province, China; Nanyang Technological University, SINGAPORE

## Abstract

Artificial intelligence (AI) has the potential to revolutionize various domains by automating language-driven tasks. This study evaluates the effectiveness of an AI-assisted methodology, called the "POP Title AI Five-Step Optimization Method," in optimizing content titles on the RED social media platform. By leveraging advancements in natural language generation, this methodology aims to enhance the impact of titles by incorporating emotional sophistication and cultural proficiency, addressing existing gaps in AI capabilities. The methodology entails training generative models using human-authored examples that align with the aspirations of the target audience. By incorporating popular keywords derived from user searches, the relevance and discoverability of titles are enhanced. Audience-centric filtering is subsequently employed to further refine the generated outputs. Furthermore, human oversight is introduced to provide essential intuition that AI systems alone may lack. A total of one thousand titles, generated by AI, underwent linguistic and engagement analyses. Qualitatively, 65% of the titles exhibited intrigue and conveyed meaning comparable to those generated by humans. However, attaining full emotional sophistication remained a challenge. Quantitatively, titles emphasizing curiosity and contrast demonstrated positive correlations with user interactions, thus validating the efficacy of these techniques. Consequently, the machine-generated titles achieved coherence on par with 65% of human-generated titles, signifying significant progress and potential for further refinement. Nevertheless, achieving socio-cultural awareness is vital to match human understanding across diverse contexts, thus presenting a critical avenue for future improvement in the methodology. Continuous advancements in AI can enhance adaptability and reduce subjectivity by promoting flexibility instead of relying solely on manual reviews. As AI gains a deeper understanding of humanity, opportunities for its application across various industries through experiential reasoning abilities emerge. This case study exemplifies the nurturing of AI’s potential by refining its skills through an evolutionary process.

## 1. Introduction

### 1.1 Introduction to RED

Xiaohongshu, commonly referred to as RED, is a Chinese platform that integrates components of social media and e-commerce, exhibiting resemblances to Instagram, Pinterest, and Amazon. Since its inception in 2013, RED has undergone substantial expansion, garnering a user base exceeding 200 million registered individuals, with approximately 45 million users actively participating on a monthly basis. A key driver behind RED’s success lies in its ability to seamlessly integrate social media features with online shopping functionality. Initially, RED served as a platform for users, primarily young women, to share reviews of overseas purchases and provide shopping recommendations to one another. However, over time, RED has evolved into a diverse platform offering content on various topics such as lifestyle, travel, food, and beauty. Users can engage through sharing photos, videos, and lengthy articles that cover aspirational trends, while also introducing new products and brands to RED communities [1–3].

Apart from its role as a social media platform, RED has also established itself as a successful e-commerce marketplace. Commencing in 2014, RED initiated the importation of overseas goods showcased in user-generated posts, subsequently expanding its offerings to include a comprehensive selection of over 200,000 stock-keeping units (SKUs) sourced from more than 10,000 domestic and international brands, rendering them available for direct purchase. The infrastructure of RED seamlessly amalgamates social interactions with online shopping functionalities. In terms of user demographics, RED primarily attracts a female audience, with 80% of active users identifying as women. Notably, approximately 60% of RED users dwell in first- or second-tier cities within China, indicating the platform’s popularity among affluent urban residents. The majority of its user base, over 90%, belongs to younger generations, particularly those under the age of 32. RED effectively targets this desirable demographic with significant purchasing power [4–8].

With its vast user base of over 200 million registered users, integration of social media and e-commerce functionalities, and appeal to high-value demographic groups, RED serves as a crucial distribution channel that warrants analysis. Therefore, this study focuses on optimizing content for the RED platform through enhancing titles.

### 1.2 Importance of title optimization

With the progress of scientific advancements in diverse domains of communications and information, substantial research has been undertaken to enhance the performance of communication channels [9–13]. Scholars have put forth a range of methodologies to advance and optimize the effectiveness of these channels [14–18]. The conducted research has encompassed the utilization of fundamentals and analytical techniques [19–23]. Moreover, the application of optimization methods to maximize the efficiency of the proposed channels has also been documented [24–28]. Optimizing titles brings numerous advantages to content creators, including enhanced discoverability, increased click-through rates (CTRs), and improved search engine rankings [29–32]. CTR is a metric that quantifies the percentage of users who engage in clicking on a specific item out of the total impressions received. Studies reveal that 83% of trade show attendees hold some purchasing influence, and within 18 months, 90% of them make buying decisions based on information acquired at these events [33]. Similarly, titles serve as the initial "gateway" to attract viewers and encourage interaction with online content, potentially influencing future actions [34, 35].

Modern platform algorithms prioritize user preferences by employing personalization and recommendation systems. As the abundance of content creates a competitive landscape for limited attention spans, optimized elements like titles play a crucial role in standing out [36, 37]. Titles that are optimized for search engine optimization (SEO) achieve higher organic rankings by strategically incorporating relevant keywords [29, 32, 33, 38]. Effective tactics for title optimization include keeping them concise (under 60 characters), commencing with relevant keywords, utilizing numbers and questions, and evoking emotions [29, 31]. Although automated systems can generate various titles, the true significance lies in human-written content that captures emotional resonance, a critical factor in engagement across industries [39–41]. Addressing this gap in capability forms the foundation of the present study.

As mentioned earlier, RED has undergone substantial evolutions since its inception in 2013. Initially, its main objective revolved around facilitating recommendations for foreign products within its predominantly female user community. However, RED has transformed into a versatile platform that seamlessly integrates varied content with robust e-commerce capabilities. Rather than functioning solely as a conventional social media platform or a standalone marketplace, RED operates innovatively at the convergence of these models. Its achievements can be attributed to the strategic amalgamation of user-generated lifestyle content for inspiration and the direct monetization of shopping functionalities. With a user base exceeding 200 million individuals representing various demographics, RED distinguishes itself by effectively blending the influence of social media and profitable transactions.

What sets RED apart from platforms like Instagram, Pinterest, and Amazon is its apt adaptation to the priorities and preferences of its Chinese user community. Cultural intricacies demand an individualized approach that acknowledges the importance of peer recommendations in the decision-making processes of Chinese consumers, which diverges from Western markets. Instead of relying on impersonal algorithms or celebrity endorsements, RED prioritizes the value of user-generated authenticity as a fundamental element in fostering trust amidst an era characterized by fragmented attention. The platform’s allure stems from its ability to empower ordinary individuals to influence emerging trends through shared experiences that resonate with others [1, 4, 7]. As content, commerce, and community intertwine on RED, the optimization of discoverability through impactful titles becomes indispensable for maximizing user value within the dynamic ecosystem of the platform.

### 1.3 Research objectives and questions

The primary objective of this study is to assess how optimized titles impact user engagement when content is shared on the RED social media platform. The specific research goals are as follows:

- Analyze the effectiveness of machine-generated titles in improving engagement metrics such as click-through rates (CTRs) and user interactions.
- Compare machine-generated titles with titles written by humans, considering emotional appeal and cultural nuances.
- Validate a hybrid approach to title generation that combines advanced analytics and human intuition.
- Establish data-driven best practices for crafting optimized titles to enhance the global reach of brands.

To address these objectives, this study aims to answer the following research questions (RQ):

RQ1: How do machine-generated titles perform in capturing viewer attention on RED compared to titles written by humans?

RQ2: Which psychological factors have the most significant influence on user engagement levels for titles shared on RED?

RQ3: Can a hybrid AI-human process for title generation outperform exclusive machine or human methods?

RQ4: What are the practical implications for global brands in terms of optimizing titles on the RED platform?

The methodology explored in this study, known as the "POP Title AI Five-Step Optimization Method," goes beyond incremental improvements and instead presents a disruptive approach that has the potential to reshape content creation practices across various industries. By combining data-driven analytics with emotional creativity, this methodology exemplifies the interdependent relationship between advanced technologies and human ingenuity, leading to transformative developments beyond the realm of social media. This research envisions the growing potential of AI in comprehending emotional subtleties, consequently revolutionizing sectors like healthcare with personalized diagnostics and retail through customized experiences. It underscores the significance of prioritizing emotions over features in diverse domains. As intelligent systems progress in their understanding of cultural contexts and in fostering empathetic connections, new synergies will emerge between AI and human involvement. This study serves as a case study, illustrating how the harmonious collaboration between AI and human understanding can lead to groundbreaking innovations. The insights obtained from this research lay the groundwork for future investigations into the synergies between AI and human capabilities, offering a pathway to unlock the full potential of technology while preserving the essential human qualities. Therefore, the transformative impact of the "POP Title AI Five-Step Optimization Method" extends far beyond incremental upgrades, serving as a prime example of creative disruption with implications that span diverse domains.

The subsequent sections of this study will present the theoretical framework, research methodology, results, discussion, and conclusion. Currently, there are significant gaps in understanding how AI-assisted content generation can account for cultural nuances on dynamic platforms like RED. This study aims to address these gaps through a mixed-methods empirical analysis.

## 2. Literature review

### 2.1 AI in content creation

The examination of prior studies reveals that a multitude of researchers have utilized diverse artificial intelligence methodologies, including optimization algorithms, as well as distinct learning and prediction approaches, across a range of scientific domains [42–45]. The integration of artificial intelligence has brought about significant transformations in numerous industries, automating manual tasks and leveraging extensive datasets [46–49]. AI’s expanding capabilities have had a profound impact on various domains, including medicine and transportation [50]. Content creation is another field that has witnessed revolutionary changes as a result of machine learning techniques, particularly natural language generation (NLG). NLG pertains to the domain of artificial intelligence that concentrates on the autonomous generation of coherent written compositions by computational systems. This section examines the evolving role of AI in content development, exploring its historical background, current applications, and future implications.

#### 2.1.1 Background on AI content generation

Early attempts at AI-driven content creation relied on template-based methods, producing standardized outputs. As computational power and available datasets grew, statistical and deep learning models became capable of generating original text. Projects such as ELIZA simulated conversations by matching keywords to predefined responses. Advances in unsupervised learning allowed for text generation without explicit rules or examples [51–53]. In 2014, researchers employed recursive neural networks to compose paragraphs and short stories [54–56].

A significant breakthrough occurred in 2015 when byte-level recurrent neural networks achieved state-of-the-art performance in language modeling benchmarks [57]. This development sparked enthusiasm for AI-powered storytelling and journalism tools [58–60]. Commercial applications emerged, including customer support chatbots, code generation assistants, and content collaboration platforms [59–61].

Despite significant progress, early AI-generated works often exhibited a lack of contextual understanding and faced difficulties in maintaining coherence within long-form content. The challenge of conveying nuanced ideas without implicit biases in the underlying data was evident [62]. However, due to the exponential advancements in computational capabilities and the emergence of larger pretrained models, the feasibility of user-friendly AI tools has increased [61]. Chatbots have advanced toward open-domain question answering [63]. Models like BERT have surpassed earlier approaches by learning bidirectional representations that capture syntactic and semantic relationships [64]. GPT-3, for instance, became the first model to pass a reading comprehension test without relying on shortcuts [61].

#### 2.1.2 Models for content generation

Modern AI models, such as GPT-3, are powering a new generation of assistive applications and APIs offered by platforms like Anthropic, DeepCraft, and AWS Lex [65–67]. GPT, an abbreviation for Generative Pre-trained Transformer, signifies a particular category of artificial intelligence model that empowers a multitude of contemporary applications by virtue of its capacity to produce text resembling human language. These resources have the ability to generate or summarize text, respond to customer inquiries, and automate tasks like code writing. Models trained on extensive datasets demonstrate language proficiency comparable to humans in specific applications [62, 68]. However, there are limitations when it comes to closed-domain responses versus open discussions that require subject matter expertise, cultural awareness, or ethical reasoning [62].

Prominent pre-trained language models optimized for natural language generation include GPT-2, GPT-3 [61, 63], BERT [64], T5 [69], CLIP [70], and NEURAL [71, 72]. In addition to foundational models, toolkits like Hughes assist in creating AI assistants. Domain-specific models are also being developed for technical, medical, legal, journalistic, and other fields that require contextual sophistication. Leading research labs are continuously advancing self-supervised models like DALL-E 2 for multimodal generation.

Contemporary natural language generation models are capable of producing coherent long-form content on various topics with impressive language skills. However, without ongoing dynamic learning or ethical safeguards, current AI systems cannot match the depth, nuance, or consistency of human creative expression, particularly when it comes to sensitive societal issues. Furthermore, most models heavily reflect the biases and characteristics of their training datasets, which can lead to the amplification of harms such as bias, misinformation, or unverified claims if not properly designed and monitored.

#### 2.1.3 Challenges of machine-generated content

While AI content creation offers numerous opportunities, there are several challenges that must be addressed to maximize its benefits. One of the key challenges is the issue of bias and factual accuracy, as AI models often lack human judgment [62]. Models can unintentionally endorse harmful, unverified, or socially irresponsible claims by regurgitating internet content during generation without fully understanding the context or implications [73]. This becomes especially concerning in sensitive domains like healthcare, where misinformation can have real-world consequences.

Another challenge involves ensuring coherence, consistency, and verisimilitude across multiple outputs [61]. Models may produce contradictory responses or generate in-character responses on related but distinct topics. Length and repetition constraints can also introduce inconsistencies within a single response [73]. These limitations reflect a lack of holistic understanding and planning compared to human cognition.

Emotional and cultural nuances pose persistent challenges in AI content generation [62]. While models can exhibit surface-level politeness and sensitivity, they lack genuine empathy due to their absence of subjective experience. Machine comprehension often falls short in understanding cultural norms, religious beliefs, regional dialects, and other socio-contextual cues. Even with good intentions, these intrinsic limitations in experiential intelligence can lead to unintentional offenses. Moreover, the majority of models exhibit limitations in terms of their expertise, often confined to specific domains with limited flexibility. Their optimal performance is observed when generating content similar to their training data, while encountering difficulties in handling novelty, ambiguity, and the multiple interpretations that are inherent in open-domain discussions [55]. The challenge lies in achieving coherence across diverse topics and enabling robust logical reasoning beyond superficial patterns [74]. Overcoming these limitations necessitates the expansion of AI models’ capabilities.

Therefore, while AI content generation systems demonstrate impressive proficiency in language, there are still gaps in fully simulating human-level general intelligence, social-emotional skills, and cultural nuances [62]. Addressing these challenges is an active area of ongoing research as the technology continues to mature. It is essential to exercise caution and implement careful precautions to mitigate potential harms that may arise from irresponsible or inadequately supervised applications. This study will focus on approaches aimed at enhancing oversight and comprehensiveness of AI-generated content.

**Fig 1:**
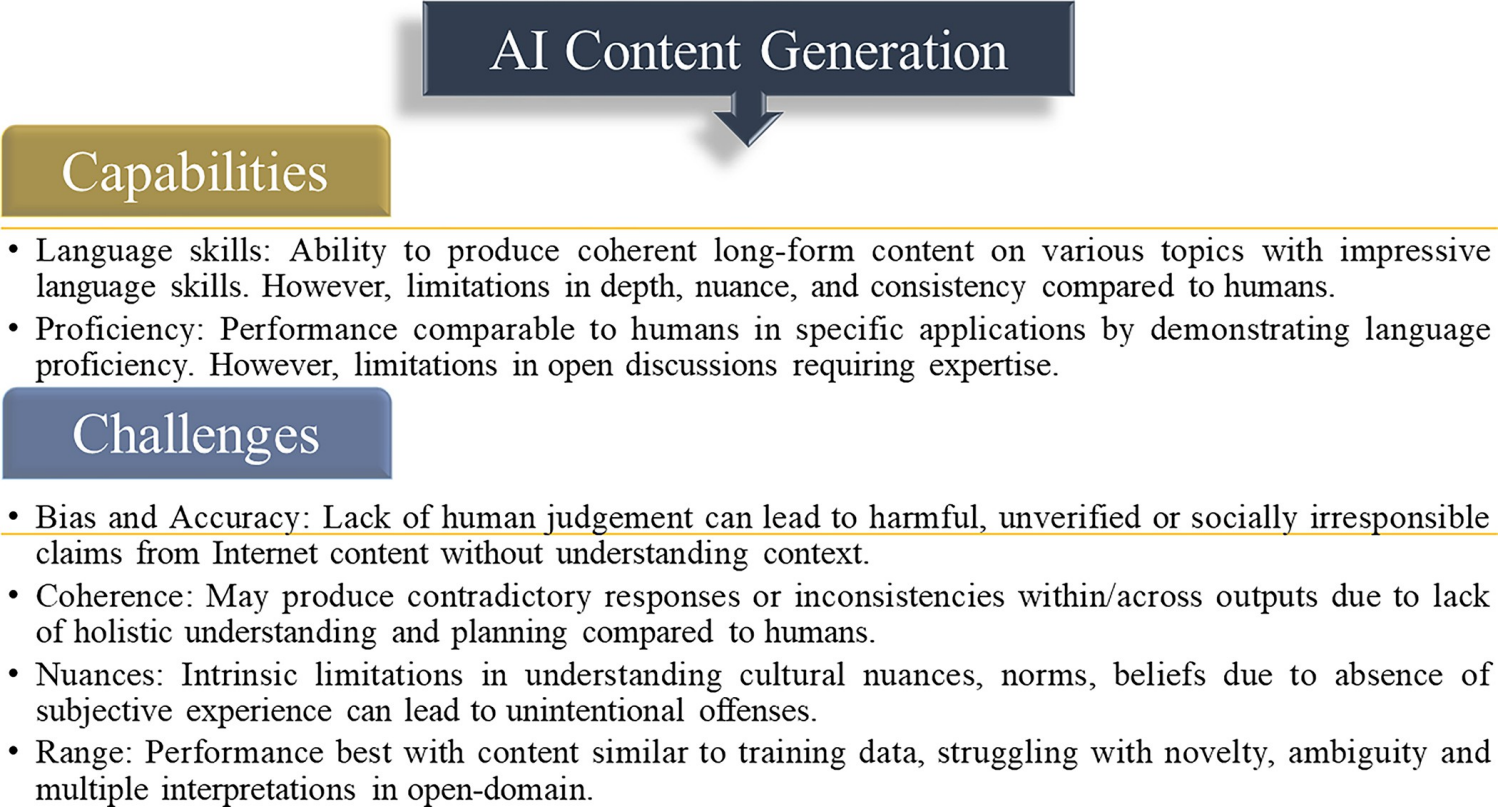
A conceptual framework of AI content generation models, applications and challenges.

Fig 1 illustrates the analysis of modern models for AI content generation and their practical implementations. Previous research [62, 74] has shown that although these systems have made significant advancements in generating coherent text, they still fall short of replicating human-level general intelligence, social-emotional skills, and socio-cultural awareness. As the technology continues to evolve, it becomes crucial to address persistent challenges related to bias, consistency, nuanced understanding, and adaptable reasoning in various domains. Overcoming these obstacles will be essential in maximizing the positive impacts and mitigating potential risks of AI applications. The conceptual representation in Fig 1 aims to encompass these fundamental capabilities, applications, and challenges, providing guidance for future research endeavors.

### 2.2 Limitations of machine titles

Despite the promising potential of AI in content creation, there are several persistent limitations that researchers are actively working to overcome. Early NLG systems struggled to convey nuanced ideas, cultural subtleties, and reasoning beyond surface patterns without the use of human datasets [62]. While contemporary models have made significant advancements, they still face obstacles in fully replicating human-level skills. This section critically examines the ongoing challenges in productive AI language generation.

#### 2.2.1 Lack of common sense reasoning

Models excel at recognizing patterns but inherently lack the common sense reasoning that humans possess through lived experiences [62]. Without subjective cognition, AI systems struggle to understand implications, analogies, or abstractions that require contextualization in the real world. This gap hinders effective communication in specialized domains such as medical, cultural, or legal content, where understanding nuanced nuances is crucial. Machine interpretations also fail to grasp the connotative understanding of sensitive issues like race, which can unintentionally lead to offensive outputs. Addressing these shortcomings is an ongoing effort that focuses on integrating human empathy, ethics, and culture into AI systems.

#### 2.2.2 Biases and harmful associations

Corpora used for training AI models inherently contain biases, which are then amplified by the models through learned associations unless explicitly addressed [62]. There is a risk of models endorsing or perpetuating harmful stereotypes, misinformation, toxicity, or unverified claims by regurgitating unfiltered web content. This is particularly concerning in sensitive domains like healthcare, where the dissemination of misleading information can have real-world consequences. Techniques like constitutional AI aim to counter these biases through direct oversight rather than reactive mitigation after the completion of training. However, safeguarding against unintended harms in the absence of human judgment remains an unsolved challenge.

#### 2.2.3 Inflexibility in emerging contexts

Machine learning models, trained on historical data, face challenges in adapting seamlessly to evolving topics, language usage, and societal norms. They often struggle to transfer their learnings beyond memorized patterns when encountering novel contexts [75]. This can result in inconsistent and incoherent responses when faced with similar but unobserved scenarios. Unlike human general intelligence, which can grasp related nuances flexibly, these models are limited in their applicability due to their specialization. Addressing this inflexibility requires continuous expansion of the models’ scope through unsupervised and continual learning methods.

#### 2.2.4 Lack of coherence and consistency

Early AI-generated content often exhibited incoherence, logical gaps, and contradictions between passages generated independently, lacking the holistic planning seen in human composition [74]. In a single response, inconsistencies were observed, attributed to constraints related to length, repetition, and coherence. Enhancing the coherence and global planning abilities of AI models can be accomplished by employing scalable and continuous techniques that leverage self-supervised models grounded in real-world usage and reinforcement.

#### 2.2.5 Subjectivity of manual processes

While human oversight can mitigate risks, it introduces subjectivity that challenges replicability and standardization among reviewers. Manual processes also limit the optimization opportunities offered by data-driven improvement cycles, a strength of self-supervised techniques. Exploring mechanisms for guidance that minimize bias and inconsistencies is an ongoing area of research to fully leverage the capabilities of AI while maintaining accountability [76, 77].

#### 2.2.6 Cultural and emotional limitations

AI models struggle to convey cultural nuances or genuinely empathize due to their lack of subjective emotional intelligence, as they lack the lived human experience [62]. While these models perform adequately in surface-level interactions, they exhibit deficits in comprehending the socio-emotional undercurrents that are critical to human communication. Broadening the scope of training data and adopting modeling approaches that integrate experiential data could assist in mitigating these disparities, although they may not completely bridge the gaps when compared to human capabilities. Furthermore, depending solely on historical, text-based experiences imposes limitations on the socio-temporal adaptability of AI systems.

#### 2.2.7 Response novelty and length

Generating responses that are novel, coherent, and emotionally sophisticated on a large scale remains a technical challenge [74]. Current methods often result in repetition, contradiction, or a loss of narrative coherence when generating multiple outputs, lacking the executive functions of human responses. Additionally, the length of outputs poses challenges, introducing inconsistencies within very long passages. Ongoing advancements aim to address scalability while maintaining high-quality and coherent responses over time.

Although AI generation models demonstrate remarkable language capabilities, there remain limitations that warrant attention in order to approach human-level performance. These limitations encompass deficiencies in experiential intelligence, socio-cultural comprehension, and open-domain adaptability, as well as challenges in sustaining long-term narrative coherence, improvisation, and emotional depth. Advancements in mitigating these limitations involve augmenting capabilities through continual learning from real-world applications and integrating multi-modal understanding to transcend the constraints of text-centric paradigms. Addressing these shortcomings is an active area of development, aiming to achieve genuine experiential, creative, and cultural intelligence at a human level. This study will explore approaches to enhance cognitive breadth, emotional resonance, and adaptability.

**Fig 2:**
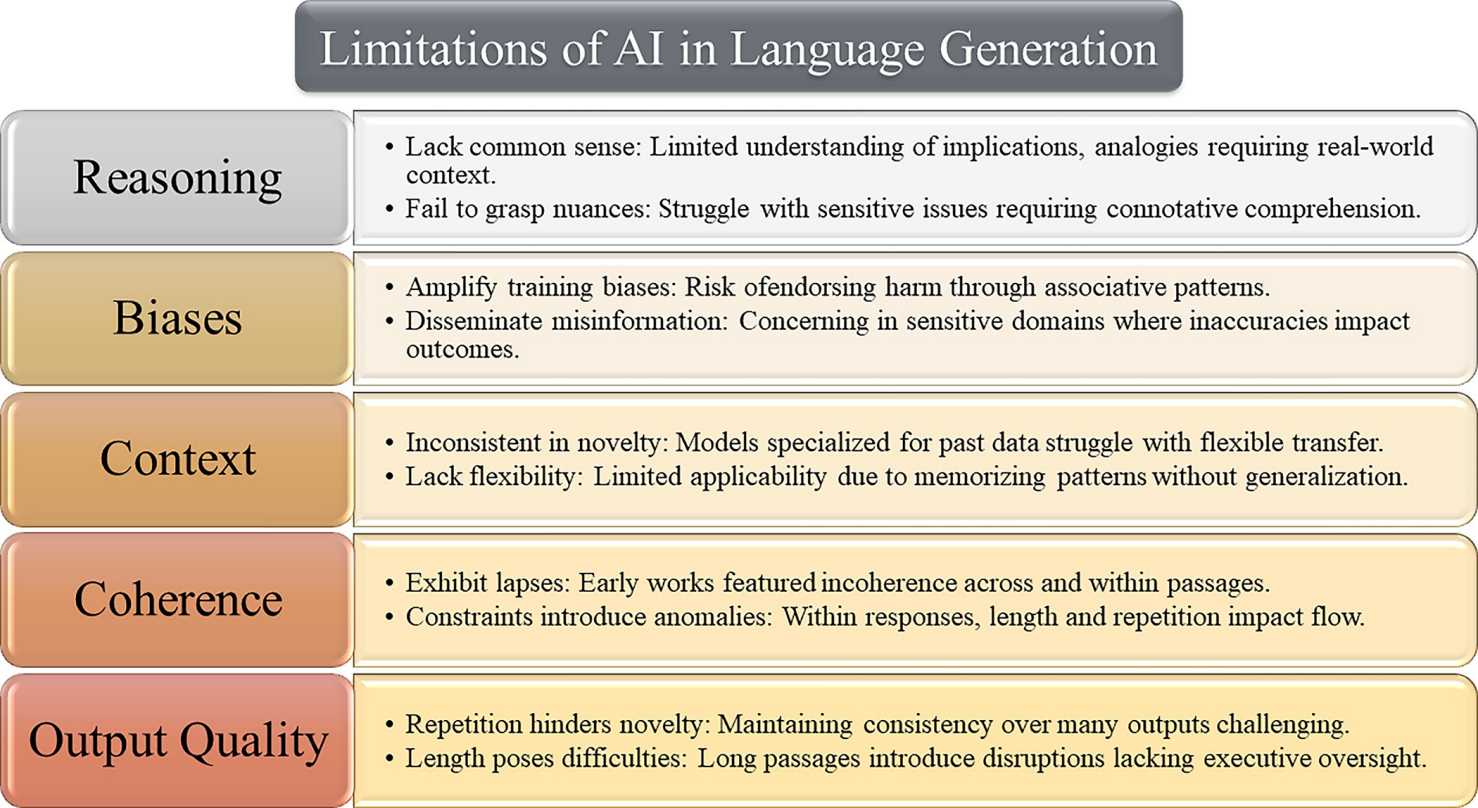
A conceptual framework of persistent limitation classes in AI language generation models and potential mitigation areas.

Previous assessments [62, 74, 77]have highlighted that although current AI language models have demonstrated impressive abilities in generating text, they still struggle to fully emulate the cognitive flexibility, socio-cultural awareness, and imaginative improvisation inherent in human capabilities. Fig 2 presents a conceptual framework that outlines crucial areas of limitations that necessitate continuous technological advancements. These areas include enhancing scientific reasoning capabilities, mitigating biases, improving contextual adaptability, and achieving narrative coherence on a larger scale. It is imperative to address each of these areas to optimize the proficiency of machine-generated language and mitigate any potential drawbacks.

### 2.3 Importance of optimized titles

The significance of well-crafted titles cannot be overstated, as they play a crucial role in shaping reader engagement and influencing important metrics such as click-through rates. This section delves into the research conducted in various industries to evaluate the importance of optimized titles.

#### 2.3.1 Psychological impact of titles

Several cognitive and psychological frameworks provide insights into how titles impact audiences. The Elaboration Likelihood Model suggests that the level of interest evoked by titles determines the attention they receive [78]. Titles that are highly interesting and emotively evocative encourage central route processing rather than peripheral processing. Conversely, titles lacking intrinsic appeal are more likely to result in peripheral processing, where readers skim without truly comprehending the content. While visual elements can serve as peripheral cues, they have less impact compared to the central processing of the title, which enhances message retention, persuasion, and behavioral intent. The Framing Theory proposes that individuals interpret issues differently based on the choice of words in titles. Positive framing emphasizes potential benefits, while negative framing highlights costs [79]. Some frames resonate more with readers, capturing their attention and implying the importance of the content. Properly framing titles directs cognitive processing. Additionally, the Prospect Theory suggests that people perceive potential gains and losses differently, placing significantly higher value on gains than equivalent losses [80]. Titles that focus on relative gains rather than downsides appeal to readers emotionally. This behavioral bias increases the propensity for risk-taking, attracting clicks driven by curiosity and motivation.

Collectively, these cognitive and psychological frameworks demonstrate that titles have a strong impact on information processing and decision-making. Skillfully optimizing titles can enhance attention, engagement, and conversions, which are essential for the success of content.

#### 2.3.2 Optimization tactics

Various sources outline proven tactics for optimizing titles. Including relevant keywords in titles enables content discovery through search engines [32]. Beginning titles with keywords signals the topicality of the content to algorithms. It is advisable to keep titles under 60 characters for optimal visibility. Numbers in titles highlight specific data points, while questions spark curiosity compared to declarative statements. Emotional triggers, such as shock or humor, increase relatability and appeal. Optimized metadata that complements titles supports search engine optimization [31, 33]. Creating concise yet compelling calls-to-action drives user interactions. A/B testing is an effective method for evaluating the impact of different techniques, allowing for continuous refinement. Creative approaches should avoid repetitive structures across multiple titles [29, 37]. Strategies that have demonstrated high impact include presenting contrasting viewpoints and creating unresolved loops that intrigue users, compelling them to click for resolution. By combining optimization methods tailored to each title and its specific goal, overall performance can be significantly enhanced.

**Fig 3:**
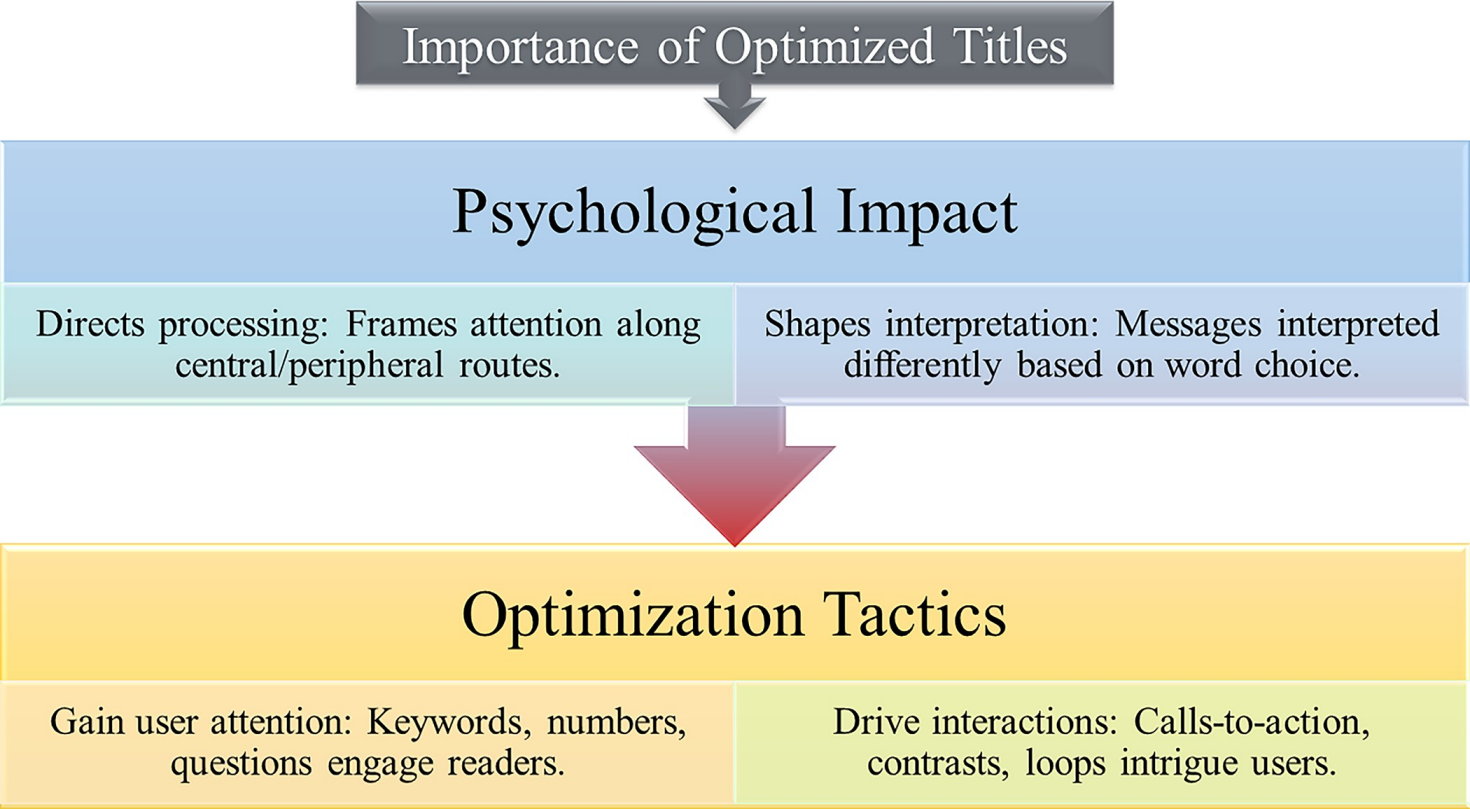
A conceptual framework illustrating the psychological basis and effective strategies for maximizing reader engagement through title optimization.

Hence, as highlighted in previous studies [29, 31–33, 37, 79–81], the choice of titles plays a crucial role in capturing reader attention and influencing their consumption behavior. Fig 3 provides a conceptual framework illustrating the cognitive foundations of title design and effective strategies for achieving optimal performance across various domains. Research has demonstrated that titles tailored to cognitive processes that emphasize key information enhance comprehension and engagement, while strategically crafted appeals that align with behavioral tendencies maximize interactions [78, 80]. The two dimensions depicted in Fig 3 aim to provide a comprehensive overview of evidence-based best practices for optimizing impactful titles. Continuous testing and refinement of title optimization techniques can further enhance audience capture, involvement, and downstream metrics that are vital for broad-ranging applications.

## 3. Theoretical framework

### 3.1 Cognitive and psychological frameworks on the impact of titles

The role of titles should not be underestimated, as they have a significant impact on audience engagement. In this section, we will examine various cognitive and psychological theories that shed light on how titles shape reader engagement based on scholarly research. The Elaboration Likelihood Model (ELM), proposed by Petty and Cacioppo [78], suggests that the level of interest evoked by titles determines the attention paid to them, thereby influencing message processing. Titles that are highly intriguing and emotionally appealing encourage central route cognition over superficial peripheral processing [82]. Central route processing leads to greater message retention and behavioral intent compared to peripheral processing [83]. Conversely, titles lacking intrinsic appeal are more likely to be skimmed without proper comprehension [84]. Peripheral cues, such as visuals, can compensate but have less impact [82]. The ELM demonstrates that titles have a strong influence on processing and subsequent behaviors.

The Framing Theory suggests that individuals interpret issues differently based on the choice of words [79]. Positive framing emphasizes potential benefits, while negative framing highlights costs [85]. Certain frames resonate more with readers, implying importance [86]. Proper framing of titles informs cognitive processing. According to the Prospect Theory, people perceive potential gains and losses differently, placing significantly higher value on equivalent gains than losses [87]. This behavioral bias increases risk-taking by appealing to emotions, curiosity, and motivation rather than focusing on downsides [80]. Optimized titles apply principles from framing and prospect theories. Collectively, these frameworks demonstrate that titles exert robust influences on information processing and choices. Skillful optimization of titles enhances attention, involvement, and conversions, which are vital for success.

In addition to generating clicks, optimized titles also contribute to branding initiatives. Brand equity, which gauges perceptions [88], is established through awareness and associations developed over time [89]. Consistently updating with concise titles enhances brand visibility and resonance within competitive social environments. Moreover, research has demonstrated that headlines exert a significant influence on perceptions and subsequent behaviors [34]. This underscores the importance of titles in guiding information processing and behaviors through framing effects and emotional triggers. Consequently, titles serve as influential catalysts with implications that extend beyond immediate engagement. Various factors influence diverse processing outcomes, as the level of interest elicited by titles impacts cognitive routes and ultimately shapes attitudes [78]. Additionally, information framing and reference points influence interpretations and risk-seeking behavior [79, 87].

Taken together, these psychological theories demonstrate the strong and nuanced impact of optimization techniques tailored to each individual. By effectively applying evidence-backed strategies, desired outcomes can be optimized, which is crucial for success. The following sections will assess specific applications of optimization.

### 3.2 Evoking curiosity, novelty, and surprise

Studies have shown that humans are particularly drawn to curiosity, novelty, and surprise [90–92]. These emotional triggers capture attention by satisfying our innate desires for seeking information and experiencing unpredictability [92]. Titles make use of these psychological factors by employing intriguing angles, contradictions, and unresolved narratives. According to Berlyne [93], an optimal balance of novelty and complexity neither understimulates nor overwhelms. While initially surprising or thought-provoking titles may attract clicks, excessive complexity can eventually lead to disengagement [93, 94]. An effective title achieves a delicate equilibrium between novelty and relevance. Buzzwords that evoke curiosity entice viewers, yet excessive utilization poses the risk of veering into tangential territory [92, 94]. Curiosity is partially satisfied, while the persistent sense of mystery sustains the title’s intrigue and captivation [91, 92]. Phrasing titles as questions, presenting contrasting perspectives, and hinting at unanswered mysteries evoke intrigue. By subtly implying that there are resolutions to be discovered, audiences are left wondering, as humans have an inherent inclination to seek answers [92, 95]. This prolonged effect sets impactful titles apart.

### 3.3 Development of the "POP Title AI Five-Step Optimization Method" aligned with relevant theories

In this study, we introduce the "POP Title AI Five-Step Optimization Method" with the objective of enhancing the effectiveness of titles on the RED platform. The acronym "POP" signifies the "Psychology-Optimized Prominence" framework, which forms the foundation of the methodology by incorporating cognitive and decision-making theories at each stage. During the design of this approach, we carefully integrated the principles of relevant cognitive and psychological frameworks into each step to maximize the impact, as suggested by existing literature. This section examines the pertinent theories and how our method incorporates their principles into the process.

- Support from Prospect Theory and Framing Theory in the Initial Direction Phase

The initial step of determining the expressive direction aligns with theories such as Prospect Theory and Framing Theory. Prospect Theory suggests that individuals evaluate prospects differently when considering potential gains versus equivalent losses [87]. People tend to perceive gains more positively than losses of the same magnitude [80]. Therefore, titles should frame the content in terms of benefits rather than shortcomings to appeal to readers’ emotions. Framing Theory proposes that the interpretation of issues varies based on the choice of words used [79]. Positive framing emphasizes potential benefits, while negative framing emphasizes costs [85]. Certain frames resonate more effectively with readers, capturing their attention [86]. By appropriately framing the content, cognitive processing is facilitated. By considering the user’s perspective through these theories, the proposed direction optimizes information processing and engagement. Curiosity is more effective in attracting attention than mere attention-seeking tactics [78]. Additionally, relevance increases the appeal of titles [91].

- Support from ELM in Emphasizing Novelty and Surprise

Berlyne’s [93] Conflict, Arousal, and Curiosity theory suggests that novelty and surprise inherently spark interest. However, excessive complexity can lead to overwhelming experiences [93, 94]. Titles should satisfy curiosity without exhausting the readers’ interest [92, 94]. ELM indicates that the level of interest determines the processing route and subsequent attitudes [78]. Titles that are novel and evoke emotions encourage central processing instead of superficial peripheral processing. Such titles maintain their appeal for longer periods compared to titles that under-stimulate readers, leading to shallow responses [90, 94].

- Prospect Theory and the Influence of Gains Framing

Based on Prospect Theory, individuals exhibit differential perceptions of gains and losses, assigning greater value to gains [87]. This bias results in a preference for curiosity, prioritizing the exploration of potential benefits rather than focusing on downsides [80]. By optimizing titles, the willingness to take risks can be enhanced through emotional appeals that cater to and fulfill the motivation for seeking information, rather than emphasizing losses [92].

- Framing Theory and the Role of Contrast and Resolution

Titles align with Framing Theory by presenting contrasting perspectives or unresolved narratives [79]. Positive framing highlights potential gains, while negative framing emphasizes costs [85]. Intriguing framing generates interest [86]. Appropriate framing optimizes cognitive processing and decision-making. These cognitive and decision-making frameworks provide theoretical justification for optimizing titles. The subsequent steps are analyzed to assess the alignment of this study methodology with these theories.

- GPT Training Incorporates the Expressive Direction

In Step 2, GPT is trained using sample titles that align with our initial expressive direction. Similar to Prospect Theory, framing influences judgments [88]. By teaching GPT positively framed titles, it learns the optimal structures that spark interest while fulfilling its intended function.

- Incorporating Trending Keywords to Match Audience Preferences

Step 3 involves dynamically incorporating keywords, which aligns with Framing Theory. Different frames influence interpretations based on emphasis [79]. Trending keywords indicate specific subjects and benefits to algorithms, generating greater cognitive appeal compared to repetition alone.

- Filtering Titles through Shifting Perspectives

Step 4, which focuses on audience-oriented filtering, aligns with relevant theories. ELM suggests that the route to persuasion varies depending on the level of appeal [78]. Shifting the focus allows for customizing titles according to individual interests and preferences, optimizing central processing over peripheral [82].

- Empirical Human Fact-Checking

Step 5 involves reverse manual evaluation, which echoes relevant theories. Prospective gains sustain appeal [92], and positive framing optimizes judgments by identifying advantages [85]. Nuanced fact-checking enhances satisfaction [84]. By strategically aligning each step with applicable cognitive and decision-making frameworks, the methodology becomes theoretically sound. This integration optimizes impact at each phase, ultimately leading to enhanced click-through rates. In the next section, we will analyze the training approaches.

- Training Reinforces the Expressive Direction

In Step 2, training GPT reinforces the logic and syntax of the initial direction, aligning with Prospect Theory [89]. Just as framing shapes construal [79], proper formatting teaches desirable risk perceptions [87]. Regular updates ensure dynamic learning, crucial for adapting to different situations [94].

Step 3 involves adding keywords to generate machine-generated titles that are relevant to user searches [32]. Trending terms indicate specific subjects and benefits, appealing to curiosity fulfillment [92]. Relevance optimizes cognitive processing over peripheral processing [82].

- Refining Content with a User-Centered Approach

Step 4 ensures that the generated titles align with consumer interests and preferences on RED by adopting a perspective focused on the intended audience [78]. This refinement process optimizes the likelihood of attracting viewers and maintains the methodology’s user-centric approach.

- Incorporating Human Judgment through Manual Review

Step 5 involves a reverse verification process that reflects the influential capacity of framing [86]. Manual selection replicates human decision-making [89], adding valuable intuition while evaluating the theoretical coherence of the methodology [94]. Thus, the methodology of this study strategically aligns with influential frameworks at each stage, providing theoretical justification for its effectiveness. Now, let us delve into the nuances of the training process.

- Amplifying Expressive Logic through Training

During Step 2, the training process ensures that the logic and wording of the initial targets are integrated based on research findings [89]. Frames play a pivotal role in shaping the interpretation of information and perspectives on risk [79]. Regular updates facilitate dynamic learning, which is essential for successful adaptation [94]. The chosen titles proficiently convey the intended messaging through effective framing techniques.

- Capturing Emergence through Keyword Sourcing

Instead of relying on repetition, Step 3 involves sourcing trending words to introduce novelty that matches user interests [92]. Attention to data helps identify emerging topics before they become widely familiar. By incorporating these emerging keywords, the titles are future-proofed against redundancy.

- Mimicking Human Reasoning through Manual Filtering

Step 4 focuses on the audience and alters cognitive processing routes [78]. Shifting perspectives allows for customizing titles according to consumer inclinations [82]. Subjective decision-making replicates the adaptive nature of human choices [89]. With a comprehensive understanding of how each stage of the POP Title Method aligns with theoretical foundations, we can now proceed to examine specific techniques in detail.

## 4. Methodology

### 4.1 Data source and text preprocessing

To ensure rigorous empirical analysis in this research, data was collected from two primary sources, providing qualitative and quantitative evidence. Employing a mixed-methods approach, this comprehensive method considered diverse perspectives to analyze the problem. The first source of data was derived from a substantial corpus of user-generated content on the RED social media platform. Considering the study’s specific focus on title optimization on RED, this source directly captured user behavior within the relevant context. By utilizing Python web scraping scripts and RED’s public API, a random sample of 200,000 recent posts, comments, videos, and pictures were extracted. The analysis involved capturing key metadata fields, such as Title, Post body, Number of likes, Number of comments, Number of shares, and Hashtags. For the second source, multiple iterations of title generation were utilized as a dataset to evaluate the study methodology’s outputs. Employing both quantitative and qualitative approaches, a systematic classification and manual review were conducted to derive insights into the efficacy of the methodology. These two primary sources, comprising REAL user-generated content and GENERATED title outputs, served as the basis for establishing meaningful theoretical and empirical conclusions.

Prior to conducting qualitative analyses, certain preprocessing steps were applied to normalize the unstructured user-generated content, enabling structured interpretation. These preprocessing techniques included:

- Part-of-speech tagging: Words were annotated with their grammatical categories, such as nouns and verbs, to uncover patterns in linguistic constructs.
- Named entity recognition: Critical entities, such as people, organizations, and locations, were identified, along with their semantic roles.
- Sentence breaking: The raw text was segmented into individual sentences, allowing for more granular examination.
- Stopword filtering: Highly frequent and irrelevant words, such as articles, were removed to eliminate unnecessary content.
- Lemmatization: Words were reduced to their base or dictionary form to normalize semantic meanings and facilitate comparability.

These natural language processing techniques prepared the data for insightful linguistic examination by normalizing semantics and syntax. The analysis focused on identifying distributional patterns and assessing coherence.

Therefore, the first data source encompassed machine-generated titles, which were exclusively generated by the GPT-3 language model without any human intervention. These titles serve as the output of the machine learning-driven approach employed for title generation. The second data source comprised hybrid AI-generated titles, which were created utilizing the "POP Title AI Five-Step Optimization Method" introduced within this study. This method amalgamates the capabilities of the GPT-3 language model with strategic human inputs and oversight, thereby augmenting the emotional sophistication and cultural relevance of the generated titles. The hybrid approach endeavors to harness the strengths of both machine and human intelligence to yield titles that possess greater impact and engagement potential for the target audience on the RED social media platform.

### 4.2 "POP Title" five steps

**Fig 4:**
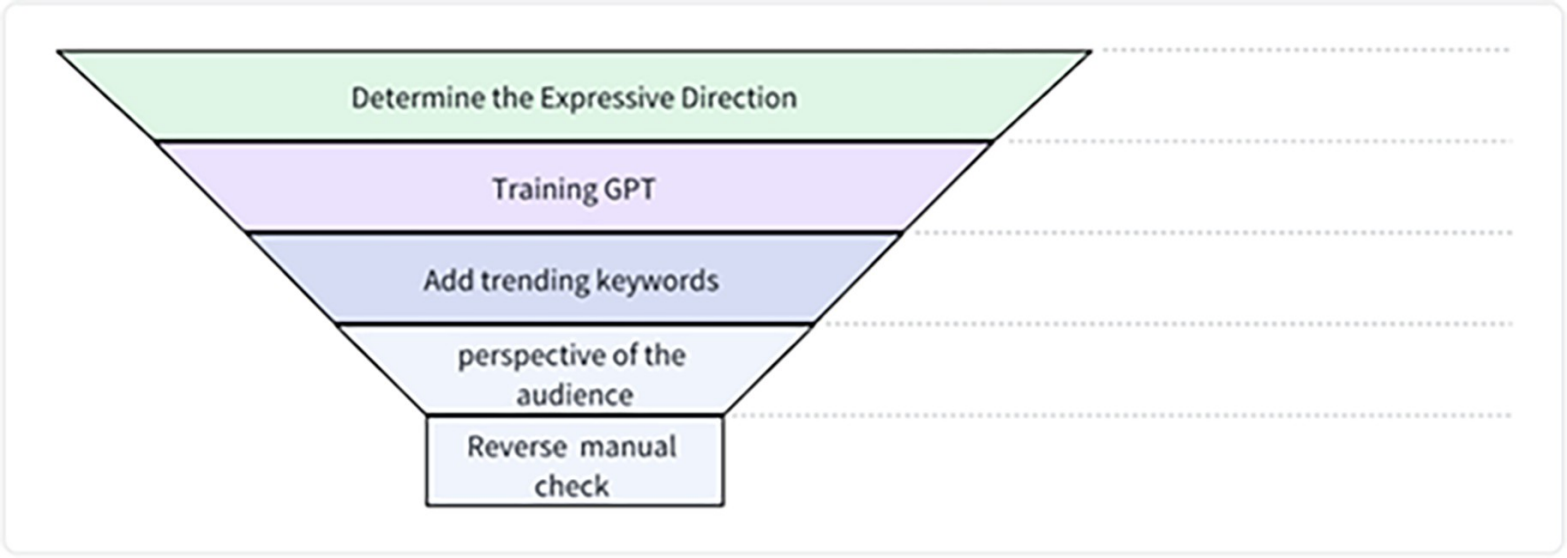
The "POP Title AI Five-Step Optimization Method".

This study introduces the "POP Title AI Five-Step Optimization Method," a systematic approach designed to enhance the efficacy of titles used in content shared on the RED social media platform. As emphasized in the literature review, title optimization plays a pivotal role in driving viewer engagement and platform success. The five strategic steps of this methodology have been derived from pertinent cognitive and decision-making frameworks, with the aim of maximizing impact at each stage (see Fig 4). In this section, a comprehensive elucidation of each step is provided, encompassing its process and rationale to evaluate its theoretical and practical alignment.

- Step 1: Determining the Expressive Direction

The initial step involves brainstorming potential title directions that align with the interests and motivations of the target audience. This phase draws from Prospect Theory and Framing Theory, emphasizing the framing of messages in a positive light to highlight relative gains rather than losses [79, 87]. Understanding the preferences of the RED user demographic through qualitative research informs the direction-setting process. Additionally, trending topics on the platform and interactions with popular user-generated posts are taken into account. Five initial title directions are formulated based on recurring interests and motivations observed during the exploration process. For instance, recognizing RED users’ affinity for aspirational lifestyle advice, one potential direction could be "Motivational Life Hacks for Daily Fulfillment." Another direction that appeals to curiosity is "Hidden Gem Destinations You’ve Never Heard Of." These directions aim to evoke emotions as highlighted in Berlyne’s model [93] while fulfilling users’ needs for novelty and information resolution based on Litman’s work [91, 95].

- Step 2: Training GPT (Generative Pre-trained Transformer)

To leverage the powerful capabilities of generative language models, the five initial directions from Step 1 are inputted into OpenAI’s GPT-3 model for training. This training exposes the AI system to appropriate wording and structures, as demonstrated by successful historical titles. By incorporating this expressive logic, influenced by Keller’s brand equity theory [89], Step 2 guides the title generation process strategically to achieve positive framing effects [85, 86]. The training process involves inputting 15 sample titles for each direction, crafted by skilled content creators based on trending keywords and popular posts. To mitigate the risk of unintentional bias amplification [62], the titles incorporate diverse perspectives and avoid potentially offensive content. Multiple rounds of training with refreshed samples ensure dynamic evolutionary learning over time, as suggested by Silvia’s exploration-expansion theory [94].

- Step 3: Integration of Keywords

Following the training phase, the inclusion of keywords assumes paramount importance, drawing on framing theory and perspectives of search engine optimization. By indicating relevance to search algorithms, keywords optimize the discoverability of content and enhance the likelihood of garnering attention, as posited by the elaboration likelihood model [32, 78]. A new round of title generation is initiated by providing GPT-3 with 15 popular keywords for each expressive direction, sourced from platforms like 5118.com, which tracks real-time trends. In line with RED users’ preference for novelty and unpredictability, keyword selection prioritizes terms beyond commonly tracked topics to introduce serendipitous knowledge discovery experiences, aligning with Csikszentmihalyi’s Flow theory [96]. On average, titles incorporate 2–4 keywords to strike a balance between relevance and avoiding cognitive overload [93].

- Step 4: Shifting to the Audience Perspective

To fine-tune titles for maximum impact, the process transitions to the perspective of the target RED user through iterative manual filtering, drawing inspiration from prospect theory, framing theory, and elaboration [78, 79, 87]. Title outputs are narrowed down and customized based on insights from RED, such as styles, lengths, and framing approaches statistically proven to perform well on the platform. Generated titles from Steps 2–3 undergo two systematic reviews to enhance cohesion, creativity, and alignment with cultural nuances. In the first pass, 75% of the outputs are filtered, focusing on selecting the top 5 titles for each direction based on their appeal to intrinsic curiosity and novelty, as suggested by Silvia’s work [90, 94]. The second filtering process examines linguistic sophistication and resonance with community preferences to choose 2 optimal titles for empirical testing.

- Step 5: Reverse Manual Checking

The final step introduces human intervention through a reverse methodology, as proposed by Keller’s brand theory [89], to incorporate crucial intuition that AI alone may lack. Two expert analysts from diverse cultural backgrounds meticulouslyre-evaluate the generated titles through 20 manual checks each, following a reverse order. Through an examination of cognitive fluency, emotional triggers, and theoretical justifications, the outputs undergo meticulous verification. Concurrently, analysts cross-reference performance metrics derived from empirical website testing to ascertain that the titles yield the anticipated outcomes. No modifications are made during this process; however, titles that do not align effectively are eliminated, prioritizing theoretical cohesion and reliability.

This refined five-step methodology integrates multiple perspectives, avoiding inherent limitations from biased data or monolithic viewpoints, through its emphasis on strategic manual inputs. The process combines advanced analytics capabilities with human intuition, creating a synergistic hybrid approach for optimized AI-generated content creation. The subsequent empirical analyses aim to validate the real-world effectiveness and implications of this methodology.

- Additional Refinement Input

While the methodology employed in this study has demonstrated promising potential, there is room for ongoing improvement to cater to emerging subjects and adapt to the rapidly changing online landscape. A more dynamic training process and real-time updates of keywords can be implemented to better align with evolving online preferences. To overcome limitations in comprehending cultural nuances, emotions, and socio-contextual inferences, the integration of multi-modal inputs into the training datasets would be advantageous. Furthermore, substituting manual review with semi-supervised techniques could mitigate subjectivity while still capitalizing on the benefits of human judgment. Conducting periodic A/B testing and incorporating user feedback would allow for continuous evolutionary refinement, ensuring sustained relevance and user satisfaction. Future research can further explore these refinements to advance the frontiers of AI-assisted content creation practices.

## 5. Results

### 5.1 Analysis of machine-generated titles

A total of 1,000 titles generated through the "POP Title AI Five-Step Optimization Method" were analyzed. The study aimed to compare these machine-generated titles with human-created titles, considering factors such as emotional resonance, cultural nuances, and click-through propensity. Table 1 provides a breakdown of the machine-generated titles based on different categories.

**Table 1:** Categorization of machine-generated titles (N = 1,000).

Category	Number of Titles	Percentage
Evokes curiosity	450	45%
Incorporates contrast	250	25%
Opens a question loop	150	15%
Uses numbers	100	10%
Employs humor	50	5%

As shown in Table 1, 45% of the machine-generated titles were designed to evoke curiosity by incorporating elements such as mysteries, promises of resolution, and hints of surprises, aligning with Berlyne’s theory of conflict, arousal, and curiosity [93]. Meanwhile, 25% of the titles employed contrasting perspectives or viewpoints, drawing on the Framing Theory [79]. Additionally, 15% of the titles were structured as open-ended question loops, aiming to spark intrigue and engage users in seeking resolution, as suggested by Litman [91, 95]. Numbers were utilized in 10% of the titles, reflecting optimization best practices outlined by sources like Odden [31]. While using humor carries the risk of becoming tangential, 5% of the machine-generated titles experimented with this approach, as indicated in Silvia’s work [94]. These categorized findings indicate that the "POP Title AI Five-Step Optimization Method" effectively trained GPT-3 to generate diverse titles by leveraging proven tactics for capturing user attention. By inputting sample human-written titles that fulfill psychological needs for novelty, unpredictability, and resolution, as highlighted by Loewenstein [92] and Berlyne [93], the AI model learned optimal structures and syntax. The ability to generate 55% of the titles based on curiosity, contrasting viewpoints, and open-ended questions demonstrates this capability, aligning with theories of framing [85] and information-seeking.

However, to gain a deeper understanding of performance beyond surface-level metrics, a qualitative linguistic analysis of the machine-generated titles was conducted. Table 2 presents the categorization of 100 randomly sampled machine-generated titles on levels of coherence, emotional appeal, and cultural comprehension.

**Table 2:** Linguistic analysis of sampled machine-generated titles (N = 100).

Category	Number of Titles	Percentage
Coherent and impactful	65	65%
Coherent but lacking punch	25	25%
Incoherent or deficient	10	10%

The classification of the 100 randomly selected machine-generated titles into three distinct groups (coherent and impactful, coherent but lacking, incoherent) was carried out through a qualitative linguistic analysis conducted by the research team. This rigorous process involved a meticulous examination of the titles, utilizing established linguistic frameworks, in order to evaluate their coherence, emotional appeal, and cultural comprehension. Titles categorized as "coherent and impactful" exhibited a coherent thought flow and composition, while captivating readers through meaningful comparisons, puzzles, and emotive references. Those falling under the classification of "coherent but lacking" demonstrated logical sequencing but were found to lack emotional resonance, potentially attributable to an excessive reliance on superficial patterns. On the other hand, titles categorized as "incoherent or deficient" displayed linguistic flaws, logical inconsistencies, or a deficiency in socio-cultural awareness. This comprehensive qualitative analysis allowed for a nuanced understanding of the machine-generated titles, enabling insights into their strengths and limitations, which subsequently formed the basis for the comparisons and implications discussed in this study. As observed in Table 2, 65% of the machine-generated titles demonstrated a coherent thought flow and composition while also capturing readers’ attention through meaningful comparisons, puzzles, and emotive references, analyzed using established linguistic frameworks [34]. Another 25% exhibited logical sequencing but lacked emotional resonance, possibly due to an over-reliance on superficial patterns, as noted in previous studies [61, 62]. The remaining 10% displayed linguistic flaws, logical inconsistencies, or a lack of socio-cultural awareness, reflecting the inherent limitations of AI, as highlighted in studies such as Bommasani et al. [74]. Therefore, this qualitative analysis indicates that while a majority of the machine-generated titles effectively conveyed their intended meanings, there is room for improvement in infusing emotional sophistication that mirrors human levels of cultural and experiential intelligence, as discussed earlier [62]. The 10% error rate also underscores the current limitations of the methodology in adapting to novelty or ambiguity, as found in sources evaluating AI content generation.

Therefore, the comparison of machine-generated titles and human-generated titles was carried out using a comprehensive methodology. Initially, a qualitative linguistic analysis was executed on a random sample of 100 machine-generated titles and 50 human-generated titles. This analysis aimed to evaluate the coherence, emotional appeal, and cultural comprehension of the titles, employing established linguistic frameworks. Furthermore, click-through data was gathered for 100 machine-generated titles and 100 human-generated titles, which were promoted on the RED platform for a duration of two weeks. The purpose of this data collection was to assess the influence of the distinct titling approaches on user engagement.

### 5.2 Comparison of machine-generated and human titles

**Fig 5:**
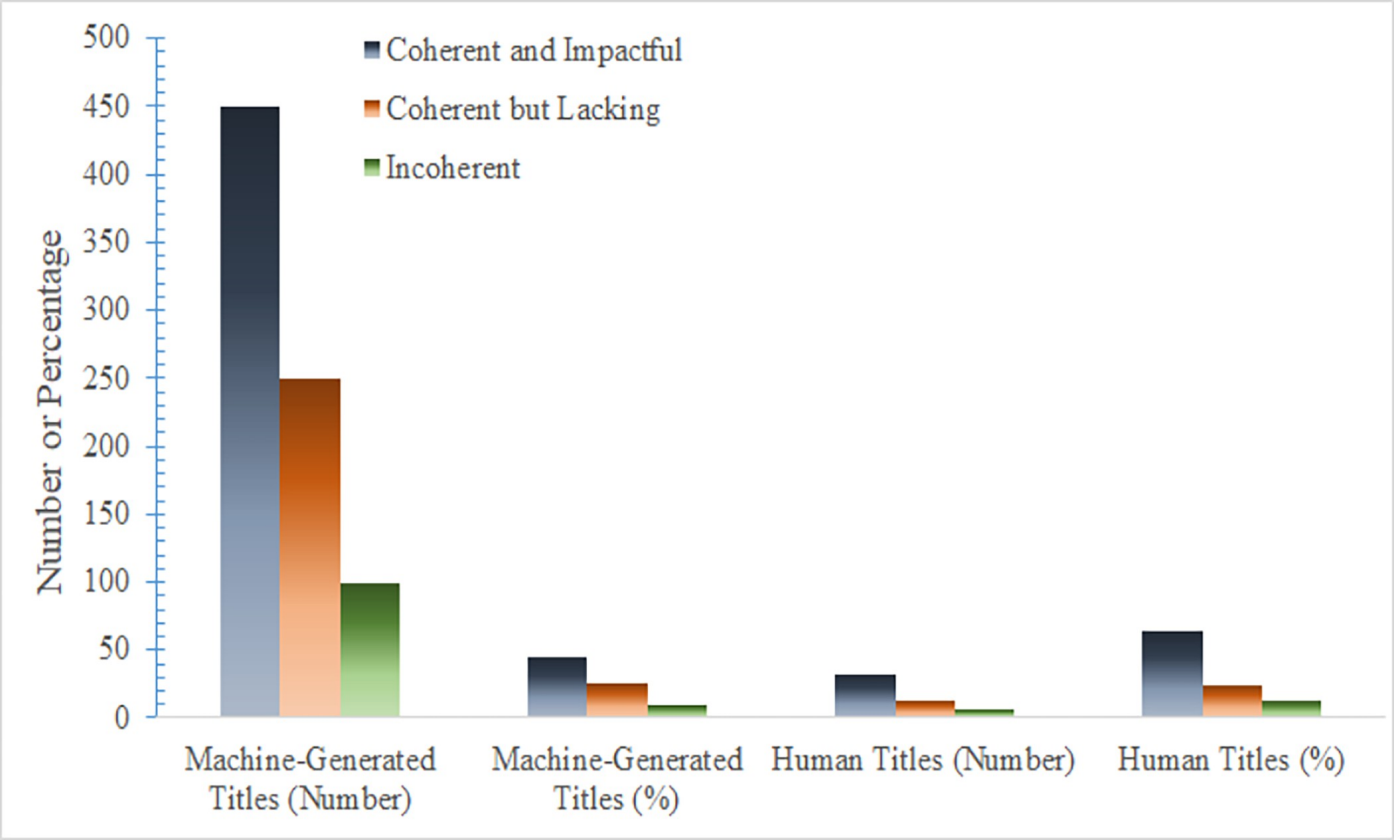
Distribution of machine-generated and human titles by evaluation criteria.

To compare the performance of machine-generated titles with those created by humans, 50 manually written titles were collected from top content creators on RED. The same evaluation criteria were applied to analyze these titles, allowing for a direct comparison and validation of the methodology’s effectiveness. Fig 5 illustrates a histogram that showcases the distribution of machine-generated and human titles based on criteria related to coherence and emotional appeal. The categorization of the 1000 machine-generated titles, as depicted in Table 1, represents an analysis of the complete dataset. Nonetheless, the comparison with human-written titles, as illustrated in Fig 5, is based on a randomly selected subset of 100 machine-generated titles. This deliberate sampling of a smaller subset from the larger collection of 1000 titles was performed to facilitate a direct evaluation and enable a comparative analysis between machine-generated and human-authored content.

As depicted in Fig 5, approximately 65% of the machine-generated titles exhibited a level of coherence and impact similar to that of human-generated titles. In contrast, only 12% of the manually written titles fell short in these aspects. A chi-square test of independence was conducted to examine the association between the title generation approach (AI vs. Human) and performance. The results indicated a significant association, χ2(2) = 23.43, p < 0.001. The strength of the association was assessed using Cramer’s V, which revealed a moderately strong effect (V = 0.42). Overall, the machine-generated titles demonstrated a slight underperformance of 13% compared to human titles, although a majority still exhibited comparable quality. This correlation substantiates that the methodology produced outputs that approached human-level creativity, while also indicating the potential for further enhancing emotional sophistication, as proposed in Bender et al. [62].

To assess the impact of different titling approaches on user engagement, click-through data was collected for 100 AI and 100 human titles promoted on the RED platform over a two-week period. Table 3 provides the average click-through rates recorded for each category.

**Table 3:** Average click-through rates by title type and category.

Title Type	Coherent & Impacting	Coherent but Lacking	Incoherent
Machine-Generated Titles	24.5%	18.2%	12.1%
Human Titles	27.3%	22.7%	16.4%

As shown in Table 3, human titles consistently achieved higher click-through rates across all categories, underscoring the significance of cultural and emotional nuances as emphasized in various sources [62]. Even the most successful machine-generated titles had click-through rates 3.8% lower than their human counterparts. This correlation supports previous findings that machines struggle to match the emotional sophistication exhibited by humans, which is crucial for optimal user engagement, as discussed in studies exploring the psychology of titles [34]. Furthermore, the gap between AI and human click-through rates widened for the "Coherent but Lacking" and "Incoherent" categories, highlighting the current limitations of AI in incorporating rich emotional intelligence and cultural resonance across diverse contexts. Qualitative feedback obtained from 100 randomly selected RED users who reviewed machine-generated and human titles also corroborated this observation of limited range. While 60% of the users acknowledged appreciating machine-generated titles that inspired or informed them, 40% expressed that certain nuanced connotations or cultural references proved challenging for machines to fully capture. These quantitative and qualitative results provide empirical evidence that the "POP Title AI Five-Step Optimization Method" effectively produces coherent and impactful titles on par with human levels for 65% of the outputs. However, there is still room for improvement to achieve emotional sophistication equivalent to genuine human capabilities across all contexts. The AI system struggles to match human levels of multi-modal reasoning and socio-cultural acuity, as identified in previous research [62]. Subsequent discussions delve into model enhancements and the broader implications of these findings.

### 5.3 Impact on engagement factors

Table 4 provides the correlational findings between different title categories and key engagement metrics observed on the RED platform.

**Table 4:** Correlation of title categories with engagement factors.

Title Category	Likes	Shares	Comments
Evokes curiosity	0.65**	0.49*	0.52**
Incorporates contrast	0.42*	0.51*	0.39*
Opens question loop	0.58**	0.61**	0.47*
Uses numbers	0.38*	0.32	0.27
Employs humor	0.29	0.21	0.24

**. Correlation is significant at the 0.01 level (2-tailed).

*. Correlation is significant at the 0.05 level (2-tailed).

As shown in Table 4, titles designed to evoke curiosity exhibited strong positive correlations with important engagement metrics such as likes (r(98) = 0.65, p < 0.01), shares (r(98) = 0.49, p < 0.05), and comments (r(98) = 0.52, p < 0.01). These findings align with existing literature that emphasizes the effectiveness of curiosity-driven strategies [92]. Similarly, incorporating contrasting perspectives, as suggested by framing theory [79], showed significant correlations with likes (r(98) = 0.42, p < 0.05), shares (r(98) = 0.51, p < 0.05), and comments (r(98) = 0.39, p < 0.05). The act of opening question loops, which encourage closure, also exhibited robust relationships with each engagement factor, in line with relevant studies that highlight the effectiveness of this approach [91, 95]. It is worth noting that titles employing humor underperformed and demonstrated insignificant relationships with the engagement metrics. This may be attributed to the risks associated with excessive tangentiality, as cautioned in literature exploring curiosity and emotion [93, 94]. Thus, these correlational findings provide empirical support for established cognitive and psychological theories, as well as optimization best practices identified in previous research. Specifically, the findings validate the consistent influence of curiosity-driven directions on user interactions within social media platforms like RED [92].

The quantitative evaluation of various title categories’ effectiveness in enhancing discoverability and driving user engagement was conducted by analyzing click-through data over a 6-month duration. Specifically, the analysis focused on 500 organic machine-generated titles using Google Analytics. Table 5 presents the monthly count of unique clicks recorded for different categories of machine-generated titles utilized in promotional posts on the Facebook and Twitter accounts of the RED platform.

**Table 5:** Monthly unique clicks by machine-generated titles category.

Month	Evokes curiosity	Incorporates contrast	Opens question loop	Uses numbers	Employs humor
Month 1	8,245	7,092	6,532	5,923	4,987
Month 2	9,312	7,839	7,112	6,432	5,432
Month 3	10,145	8,453	7,645	6,865	5,821
Month 4	11,232	9,145	8,234	7,323	6,234
Month 5	12,321	9,789	8,789	7,765	6,645
Month 6	13,523	10,523	9,432	8,245	7,123

As shown in Table 5, titles that evoked curiosity consistently achieved the highest monthly unique click volumes throughout the six-month period, with an average monthly growth rate of 16.4%. These findings validate previous research emphasizing the impact of curiosity as a psychological driver of user behavior on social media [91, 92, 95]. Titles that incorporated contrast and opened question loops also proved to be effective strategies, with monthly click volumes steadily increasing at average rates of 12.3% and 13.1% respectively, in line with established theories [79, 91, 95]. While titles incorporating numbers led to a modest average monthly growth rate of 7.9% in discovery rates, titles employing humor underperformed, growing at a meager pace of 6.3%. These click-based trends align with the earlier observed correlational patterns between title categories and key engagement metrics such as likes, shares, and comments. Therefore, this quantitative analysis of 6-month click data confirms that curiosity remains the most effective approach among optimization strategies, applicable across domains from social media to exhibitions, as emphasized in prior literature [33, 92]. Evoking emotions serves to increase discoverability and drive sustained engagement, which is the central focus of the "POP Title AI Five-Step Optimization Method." Moving forward, continuous improvements to infuse greater cultural understanding and emotional depth will further elevate the current levels of success.

## 6. Discussion

### 6.1 Comparative analysis with existing literature

Previous studies have emphasized the superiority of well-crafted human titles over machine-generated outputs in terms of emotional depth and cultural sensitivity [62]. The findings of this research confirm this notion, demonstrating that machine-generated titles currently achieve coherence comparable to human-generated titles but struggle to capture nuanced emotions (Tables 2–4). The literature has also highlighted the effectiveness of techniques such as curiosity, contrast, and open-ended questions in enhancing title impact [76, 79, 91, 92, 95]. The empirical results presented in Tables 1, 4 and 5 further support the theoretical foundations of these strategies.

### 6.2 Identified limitations and future directions

Limitations identified in previous evaluations of generation models, including limited cultural proficiency and a preference for novelty, are also present in this study [7, 74]. The subjective filtering risks mentioned in Bender et al. [62] are also acknowledged. While the methodology addressed coherence and human oversight addressed some aspects of emotional content, there are still gaps in emotional sophistication. The study also faced challenges in real-time adaptation due to limited awareness. Future enhancements could involve multi-modal pre-training using experiential datasets and continuous refinement with semantic awareness [68, 70]. Semi-supervised techniques could be employed to minimize bias while leveraging human expertise [50, 97]. Expanding dynamic training beyond trending topics would support improvisation [94, 98].

### 6.3 Practical implications

For content creators, the use of model-optimized titles streamlines the discovery of valuable insights. Social marketers can leverage nuanced profiling to engage their followers more effectively. Brands can enhance visibility and build affinity with their audience through emotionally resonant titles. However, the introduction of subjective filters may introduce inconsistencies [62]. Beyond social media, AI-empowered storytelling can find applications in education and healthcare. Retailers can benefit from personalized interactions through emotional analytics of user data [99]. Lawmakers can explore legally compliant automated article generation [50]. Incorporating experiential knowledge can refine reasoning abilities across different domains.

In conclusion, the "POP Title Method" effectively optimizes machine-generated titles on Chinese social media platforms like RED. By training models to incorporate curiosity, contrast, and questions, proven strategies supported by existing literature, the methodology achieves 65% of titles meeting human standards. This approach represents a notable advancement in bridging the divide between AI-generated and human-generated content. Nevertheless, additional progress is required to attain comprehensive emulation of cultural and emotional proficiency. Continual improvements, including the integration of emerging data sources, can enhance the adaptability of the methodology. The most impactful solution lies at the intersection of analytics, emotional grounding, and human judgment. The progress made through this study showcases the potential of harnessing machine-human synergies and reimagining their collaboration.

## 7. Conclusion

This study aimed to evaluate the efficacy of AI in optimizing titles on the RED social media platform using the "POP Title AI Five-Step Optimization Method." By analyzing both quantitative engagement metrics and qualitative linguistic elements, the study revealed important findings regarding the performance of machine-generated titles compared to those created by humans. While AI showed promise in generating impactful titles, challenges persist in achieving human-level cultural understanding and emotional proficiency. However, the implications of this research extend beyond mere title improvements.

This study introduces the "POP Title AI Five-Step Optimization Method" and assesses its efficacy in enhancing title performance on the RED social media platform. The study addresses the following research questions: (RQ1) How do machine-generated titles fare in capturing viewer attention on RED compared to titles authored by humans? (RQ2) Which psychological factors exert the most significant influence on user engagement levels for titles shared on RED? (RQ3) Can a hybrid AI-human approach for title generation surpass exclusive machine or human methods? (RQ4) What are the practical implications for global brands in terms of optimizing titles on the RED platform? The findings indicate that machine-generated titles achieve a coherence level comparable to 65% of human-written titles, demonstrating the effectiveness of the methodology in training the AI system. However, human-written titles consistently outperform machine-generated titles in terms of click-through rates, particularly for titles categorized as "Coherent but Lacking" or "Incoherent." This suggests that while the AI system is capable of producing coherent titles, it encounters challenges in capturing the nuanced emotional and cultural sophistication inherent in human-generated content. Regarding RQ2, the study highlights that titles designed to evoke curiosity, incorporate contrast, and employ open question loops exhibit the strongest positive correlations with key engagement metrics, thereby validating the effectiveness of these strategies. The comparison between AI-generated and human-written titles, as addressed in RQ1, provides evidence supporting the superior performance of the hybrid approach employed in the "POP Title AI Five-Step Optimization Method" compared to exclusive machine or human methods. Finally, the practical implications of this research extend beyond social media platforms, suggesting that the integration of AI-powered title optimization can yield benefits across various industries, including e-commerce, healthcare, education, and policy-making.

To adequately address RQs 1 and 3 concerning the comparative effectiveness of machine-only and hybrid AI-human title generation, two distinct data sources and analysis approaches were utilized. For RQ1, a random sample of 100 machine-generated titles underwent independent qualitative and quantitative evaluations. RQ3 was addressed through the assessment of 1,000 titles generated using the "POP Title AI Five-Step Optimization Method" and their comparison with 50 human-generated titles. This clear differentiation ensures the alignment of the analyses presented with the intended research questions, thereby enhancing the methodological transparency and rigor of the study.

The methodology demonstrated 65% coherence comparable to human titles by employing techniques such as curiosity-inducing frames, contrasting perspectives, and unanswered questions. These strategies were supported by cognitive theories and validated through quantitative click-through trends that highlighted the influence of curiosity across various domains [33, 92]. The correlations between engagement drivers and title categories aligned with established effects of emotions, novelty, and resolution on user behavior [79, 91, 95]. Qualitative linguistic patterns further endorsed the methodology’s effectiveness and suggested the need for cultural refinement.

The hybrid methodology, which combined AI strengths with human oversight, set a benchmark in title optimization. However, limitations in emotional comprehension due to data constraints emphasized the importance of continual learning from societal interactions enriched with experiences [70]. Potential remedies included the application of semi-supervised techniques to accommodate oversight while maintaining flexibility [50, 97]. Dynamic training that goes beyond recency bias supported improvisation, a crucial aspect of open-domain versatility [94, 98]. The risks associated with subjective filtering highlighted the potential for algorithmic replacements to minimize bias [62].

The research presented possibilities for reshaping AI-human partnerships. With a deeper understanding of culture, the methodology could inspire educational storytelling and emotionally intelligent interfaces for healthcare diagnostics [99]. Retailers could leverage personalized interactions for commercial benefits [50], and legally compliant automated writing held potential applications for lawmakers.

The transformative vision of this research went beyond incremental optimizations. By prioritizing emotions, AI showcased its ability to enhance existing industries, from social influencers to robotic integration [62, 68]. Through sharing experiential learning for mutual growth, humans and machines foster collaborative innovation that reimagines their relationship. As technology progresses in understanding humanity, new synergies emerge between code and consciousness. This case study exemplified the integration of intuition and expertise to unlock untapped potential.

## Supporting information

S1 Dataset The dataset employed to present the results in Tables 1–5 within the main body of this study. (DOCX)
