# Supplementary material for: Beyond algorithms: The human touch machine-generated titles for enhancing click-through rates on social media
Source: PLoS One. 2024 Jul 12;19(7):e0306639. doi: 10.1371/journal.pone.0306639 (PMC11244827; doi:10.1371/journal.pone.0306639)
Supplement: S1 Dataset — (DOCX) [file pone.0306639.s001.docx]

This documentation encompasses the dataset compiled and examined during the research. The study employed a mixed-methods approach to collect both quantitative and qualitative data from two primary sources:

- User-generated posts and interactions on the RED social media platform.
- Outputs produced by applying the "POP Title AI Five-Step Optimization Method."

The collected data was then categorized and subjected to statistical analysis to identify patterns and correlations among various variables. Below, you will find five datasets that correspond to the results tables presented in the study. These datasets ensure the protection of anonymity and confidentiality for all individuals and organizations involved.

The subsequent section provides the complete datasets analyzed and presented in Tables 1 to 5, which evaluate the effectiveness of AI-generated title optimization using the outlined methodology. For a comprehensive understanding of the research methodology, analysis techniques, theoretical frameworks, limitations, and conclusions, please refer to the original study manuscript.

| Table 1 Dataset | | |
| --- | --- | --- |
| Category | Number of Titles | Percentage |
| Evokes curiosity | 450 | 45% |
| Incorporates contrast | 250 | 25% |
| Opens a question loop | 150 | 15% |
| Uses numbers | 100 | 10% |
| Employs humor | 50 | 5% |

The row labels indicate the different categories that the AI titles were classified into based on prominent elements observed through analysis.

The second column "Number of Titles" shows the number of titles from the total 1000 AI titles that fell under each category.

The third column "Percentage" shows the percentage of titles from the total 1000 AI titles that each category represents.

The percentages were calculated based on the number of titles in each category divided by the total number of titles, which is 1000.

| Table 2 Dataset | | |
| --- | --- | --- |
| Category | Number of Titles | Percentage |
| Coherent and impactful | 65 | 65% |
| Coherent but lacking punch | 25 | 25% |
| Incoherent or deficient | 10 | 10% |

The row labels indicate the different categories that the 100 randomly sampled AI titles were classified into based on a linguistic analysis evaluating coherence, emotional appeal, and cultural comprehension.

The second column "Number of Titles" shows the number of titles from the total 100 AI titles that were categorized under each classification.

The third column "Percentage" shows the percentage that each category's number of titles represents out of the total 100 AI titles that were sampled and analyzed.

The percentages were calculated based on the number of titles in each category divided by 100, which is the total number of AI titles that were sampled.

65 titles were found to be coherent and impactful, representing 65% of the total. 25 titles were coherent but lacking, representing 25%. 10 titles were incoherent or deficient, representing 10%.

| Table 3 Dataset | | | |
| --- | --- | --- | --- |
| Title Type | Coherent & Impacting | Coherent but Lacking | Incoherent |
| AI Titles (Average CTR) | 24.5% | 18.2% | 12.1% |
| Human Titles (Average CTR) | 27.3% | 22.7% | 16.4% |

The row labels indicate whether the title was an AI-generated title or Human-generated title.

The column labels represent the different categories that the 100 AI titles and 100 human titles were evaluated under based on coherence and emotional appeal.

The cells within the table show the average click-through rates (CTRs) recorded for titles that fell under each category type.

For AI titles:

- Coherent & Impacting titles had an average CTR of 24.5%
- Coherent but Lacking titles had an average CTR of 18.2%
- Incoherent titles had an average CTR of 12.1%

For Human titles:

- Coherent & Impacting titles had an average CTR of 27.3%
- Coherent but Lacking titles had an average CTR of 22.7%
- Incoherent titles had an average CTR of 16.4%

| Table 4 Dataset | | | |
| --- | --- | --- | --- |
| Title Category | Likes | Shares | Comments |
| Evokes curiosity | 0.65** | 0.49* | 0.52** |
| Incorporates contrast | 0.42* | 0.51* | 0.39* |
| Opens question loop | 0.58** | 0.61** | 0.47* |
| Uses numbers | 0.38* | 0.32 | 0.27 |
| Employs humor | 0.29 | 0.21 | 0.24 |

The row labels indicate the different categories that the AI titles were classified into.

The column labels represent the key engagement metrics of Likes, Shares, and Comments that were analyzed based on the categorization.

The cells within the table show the correlation coefficient between each title category and the respective engagement metric.

**. Correlation is significant at the 0.01 level (2-tailed).

*. Correlation is significant at the 0.05 level (2-tailed).

| Table 5 Dataset | | | | | | |
| --- | --- | --- | --- | --- | --- | --- |
| Title Category | Month 1 | Month 2 | Month 3 | Month 4 | Month 5 | Month 6 |
| Evokes curiosity | 8,245 | 9,312 | 10,145 | 11,232 | 12,321 | 13,523 |
| Incorporates contrast | 7,092 | 7,839 | 8,453 | 9,145 | 9,789 | 10,523 |
| Opens question loop | 6,532 | 7,112 | 7,645 | 8,234 | 8,789 | 9,432 |
| Uses numbers | 5,923 | 6,432 | 6,865 | 7,323 | 7,765 | 8,245 |
| Employs humor | 4,987 | 5,432 | 5,821 | 6,234 | 6,645 | 7,123 |

The row labels indicate the different categories that the 500 AI titles optimized based on.

The column labels represent the individual months that the click data was analyzed over.

The cells within the table show the monthly unique click volumes recorded for each category over the 6 month period.
